# Cancer incidence in kidney transplant recipients: a study protocol

**DOI:** 10.1186/1471-2407-9-294

**Published:** 2009-08-22

**Authors:** Salvador Pita-Fernandez, Francisco Valdes-Cañedo, Sonia Pertega-Diaz, Maria Teresa Seoane-Pillado, Rocio Seijo-Bestilleiro

**Affiliations:** 1 Clinical Epidemiology and Biostatistics Unit, University Hospital Complex of A Coruña, Hotel de Pacientes 7a Planta, As Xubias, 84, 15006 A Coruña, Spain; 2 Department of Nephrology, University Hospital Complex of A Coruña, Spain

## Abstract

**Background:**

Different publications show an increased incidence of neoplasms in renal transplant patients. The objective of this study is to determine the incidence of cancer in the recipients of renal transplants performed in the A Coruña Hospital (Spain) during the period 1981–2007.

**Methods/Design:**

During the study period 1967 kidney transplants were performed, corresponding to 1710 patients. Patients with neoplasms prior to the transplant will be excluded (n = 38). A follow-up study was carried out in order to estimate cancer incidence after transplantation.

For each patient, information included donor and recipient characteristics, patients and graft survival and cancer incidence after transplantation. Incident cancer is considered as new cases of cancer after the transplant with anatomopathological confirmation. Their location will be classified according to the ICD-9.

The analysis will be calculated using the indirect standardisation method. Age-adjusted cancer incidence rates in the Spanish general population will be obtained from the Carlos III Health Institute, the National Epidemiology Centre of the Ministry of Science and Technology. Crude first, second and third-year post-transplantation cancer incidence rates will be calculated for male and female recipients. The number of cases of cancer at each site will be calculated from data in the clinical records. The expected number of cancers will be calculated from data supplied by the Carlos III Health Institute. For each tumour location we will estimate the standardized incidence ratios (SIRs), using sex-specific cancer incidence rates, by dividing the incidence rate for the transplant patients by the rate of the general population. The 95% confidence intervals of the SIRs and their associated p-values will be calculated by assuming that the observed cancers follow a Poisson distribution. Stratified analysis will be performed to examine the variation in the SIRs with sex and length of follow-up.

Competing risk survival analysis methods will be applied to estimate the cumulative incidence of cancer and to identify variables associated to its occurrence.

**Discussion:**

Information about cancer incidence in kidney transplant patients could be useful to adapt the guidelines on post-kidney transplant follow-up on tumour screening, and evaluate the impact of intervention measures for the prevention of cancer in these patients.

## Background

Cardiovascular illnesses and neoplasms are the two main causes of death with normal function of the graft in the long-term follow-up of patients who have received kidney transplants [1,2]. The Australian-New Zealand register even suggests that the second factor could be growing at a faster rate than the first [3]. The presence of neoplasms is a major threat and cause of morbidity in kidney transplant patients.

According to data published in other countries, the accumulated incidence of neoplasms can reach 20% 10 years from the transplant [4] and nearly 30% after 20 years [5,6]. The rate of expected cancers compared to those which are observed varies in the different registers. On average, it is estimated that the incidence of cancer in patients who have received kidney transplants is 3 times higher than that for the general population. By localizations, this ratio can reach an incidence rate of between 8 and 14 times more for kidney cancer in transplant patients, and an incidence of between 65 and 92 times more of non-melanoma skin tumours [4,5,7-11]. In the largest study on the incidence rates of malignancies among first-time recipients of deceased or living donor kidney transplantation (n = 35765) the rates for most malignancies are higher after kidney transplantation compared with the general population [7]. Similar results were observed in studies from five national tumour registries in Denmark [11], Finland [12], Sweden [13], Australia [14], and Canada [15] with a total sample size of 31,977 transplant recipients.

Several factors may influence in the pathogenesis of tumours after transplantation: chronic uraemic status, cumulative exposure to immunosupression, and certain drugs which can be carcinogenic through independent mechanisms or as a result of immunosupression and viral infections. Some authors suggest that immune deficiency, rather than other risk factors for cancer, is responsible for the increased risk [16].

The use of immunosuppressive agents to prevent allograft rejection increases the long-term risk of malignancy compared with that of the general population. In this case, cyclosporine and tacrolimus have been identified as oncogenes in themselves, through a mechanism which involves TGF-β [17,18]. However, the data published to date do not make it possible to obtain conclusive results.

Despite the increased risk, little is known about the overall cancer prognosis, screening, treatment strategies, and effectiveness in this population [19], and strategies to understand and minimize the risk of developing malignancy in the transplant population are needed [20]. Some authors describe this uncertainty as an "unmet medical need" [21].

Most of the information regarding the incidence of post-transplant tumours comes from multi-centre registries, in which the data gathering process may be incomplete, and which may lead to an underestimation of the true incidence of malignant cancers. Also, it is difficult to estimate the incidence of the majority of tumours and their associated factors based on studies carried out in a single hospital, which normally involve a small number of patients.

Knowledge of the true incidence of cancer in patients who have undergone kidney transplants in our surroundings could alert us to the need to establish tumour screening policies which permit early intervention, as has been established in the European Guidelines for kidney transplant follow-up [22-24]. Promoting healthy lifestyles and carrying out regular tests on patients that permit the early diagnosis of tumour complications could contribute towards palliating the morbidity-mortality of these patients.

The aim of this study is to establish the incidence of cancer in recipients of renal transplants performed in a hospital in A Coruña (Spain) during the period 1981–2007 compared to that experienced by the general population. This centre, the University Hospital Complex of A Coruña, has the same level of activity as the national average, at around 50 interventions per million population per year.

## Methods/Design

An observational prospective follow-up study with a retrospective component, carried out in the health district of A Coruña (northwest Spain) during the period 1981–2007. During that period 1967 kidney transplants were performed in the University Hospital Complex of A Coruña, which corresponded to 1710 patients. Patients with pre-transplant neoplasms will be excluded from the analysis (n = 38). A follow-up study was designed in order to estimate cancer incidence after kidney transplantation. This sample size would make it possible to detect relative risks of ≥1.2 estimating an exposed proportion of 35% and a proportion of censured observations of 40%, with a security of 95% and a statistical power of 80%.

Patients who had received transplants were identified through the hospital's transplant registry. For each patient, information includes donor and recipient characteristics, patient and graft survival and cancer incidence after transplantation. The variables are summarized in the Appendix.

The study was approved by the region's Ethics Committee for Clinical Research (CEIC Galicia) and written informed consent was obtained from all of patients who have participated in the study.

### Follow-up period

The period of follow-up for each patient starts on the day of transplantation and continues until death or last reported contact. Following the methodology used in similar studies, patients will not be removed from the analysis at the time of graft failure for several reasons [14,25]: (i) cancer risk is likely to persist despite graft failure or, otherwise, the risk of malignancy is unlikely to return to pre-transplant levels following graft failure, (ii) previous studies have established that dialysis is also associated with increased cancer risk in comparison with the general population, and (iii) we think it is important to inform clinicians and patients about cancer risks following transplantation.

### Determining the incidence of cancer after transplantation

Incident cancer is considered as new cases of cancer which occur after the transplant and which have anatomopathological confirmation. Their localization will be classified according to the International Classification of Diseases-9 (ICD-9). Observed cancers are those reported at any time after the date of first transplantation, and include those that occurred after a subsequent transplant, following a "once transplanted always transplanted" rule [5].

### Comparing cancer after transplantation with cancer in the Spanish population

The analysis will be calculated using the indirect standardisation method. Estimates of age-adjusted cancer incidence rates in the general population of Spain will be obtained from the Carlos III Health Institute, the National Epidemiology Centre of Spain's Ministry of Science and Technology.

Crude first, second and third-year post-transplantation cancer incidence rates will be calculated for male and female recipients. The number of observed cases of cancer at each site will be calculated from data in the clinical records.

The expected number of cancers will be calculated from data supplied by the Carlos III Health Institute. For each tumour location we will estimate the standardized incidence ratios (SIRs) of cancer, using sex-specific cancer incidence rates, by dividing the incidence rate for the transplant patients by the rate of the general population. The 95% confidence intervals of the SIRs and their associated p-values will be calculated by assuming that the observed cancers follow a Poisson distribution. Stratified analysis will be performed to examine the variation in the SIRs with sex and length of follow-up.

### Estimating the incidence of cancer over time

Competing risk survival analysis methods will be applied to estimate the cumulative incidence of developing cancer over time from kidney transplantation.

This method allows for the fact that a patient may experience an event which is different from the event of interest. These events are known as competing risk events, and may preclude the onset of the event of interest, or may modify the probability of the onset of the event of interest. In particular, a transplanted patient may die or lose the graft without developing any kind of cancer. In a Kaplan-Meier estimation approach, these persons would be treated as censored and would be eliminated from the risk set. This could lead to misleading results, as it is based on the assumption that censoring is "non-informative", meaning that a censored patient has the same risk of developing cancer as those who have complete follow-up. This is not the case in patients who die before without developing cancer, as they are no longer at risk.

Occurrence of cancer will be the event of interest. Any other event, such as death of graft failure, will be considered competing events. Estimates of cumulative incidence functions will be calculated based on the two-step process developed by Kalbfleisch and Prentice [26]. In the first step, we will calculate the Kaplan-Meier estimate of the overall survival from any event. In the second step, the conditional probabilities of developing cancer will be calculated. The cumulative incidences will be estimated using these probabilities. Differences between the cumulative incidence functions according to patient characteristics will be tested for statistical significance using the method developed by Gray [27]. The cumulative incidence function regression model of Fine and Gray [28] will be used for multiple regression analyses.

Statistical analysis will be performed by using the R statistical package (version 2.9. 2009; The R Foundation for Statistical Computing) [29] and EPIDAT statistical software (version 3.1, 2006; Dirección Xeral de Saude Pública, Organización Panamericana de la Salud) [30]. P-values of < 0.05 are considered statistically significant.

## Discussion

This study will provide us with information on the risk of developing cancer in kidney transplant patients, and will allow us to increase the level of vigilance over the incidence of cancers in this population, especially for tumours for which there are no population-based screening programmes.

The interval between transplantation and diagnosis of cancer is time dependent, so the follow-up period will let us know if the incidence is modified with length of follow-up. Even though there is variability dependent on the type of tumour and age of the patient, it has been described that the average latency period is approximately three to five years after transplantation [31-34].

Furthermore, this study will provide us with information on the variables associated with the presence of cancer in kidney transplant patients, and allow us to adapt the European Guidelines on post-kidney transplant follow-up on tumour screening to our environment[35].

Although the information will be taken from hospital records, which could lead to a bias in the information, the characteristics of these patients mean that they are subjected to more exhaustive follow-up than normal, not only during the immediate post-transplant period, but also during the years after the transplant. During the first year after the transplant they are seen at least every 3 months, every 6 months from one year after the transplant, and after 10 years are seen at least once a year. This fact would minimize any possible bias in the information. The use of data from a single centre could also hinder the calculation of the incidence of less frequent tumours. It is also true that as indicated in a number of publications, neither is the incidence of cancer in populational registries of transplant patients free from errors, as the reporting of cancer to the registry is often incomplete, and this could lead to an underestimation of the incidence [7].

Spain has the highest cadaver donor rate in the world, and is the leading country at international level in terms of performance in renal transplantations per million population (pmp) [36]. The hospital in which we are going to carry out this study, the University Hospital Complex of A Coruña, is located in the Autonomous Region of Galicia, which has the same level of activity as the national average, at around 50 interventions per million population per year [37].

Little is known about the advantages and disadvantages of cancer screening, treatment effectiveness, and overall cancer prognosis in renal transplant recipients [38].

This study will allow us to know the scale of the problem in kidney transplant patients, and to evaluate the impact of intervention measures for the prevention of cancer in these patients in the future.

## Competing interests

The authors declare that they have no competing interests.

## Authors' contributions

SPF was involved in the conception of the study and participation in its design. FVC was involved in the conception and coordination of the study. SPD and MTSP participated in the design of the study. RSB was involved in acquisition of data and help in drafting the manuscript. All authors have read and approved the final manuscript.

## Appendix

### Study measurements

#### PATIENT

Date of transplantation

Age at transplantation

Gender

Body mass index

Primary renal disease: diabetic nephropathy, glomerulonephritis, hypertensive and arteriopathic conditions and others.

Prior time spent on dialysis, and dialysis modality

Pre-transplant cancer

Smoking status at transplant and during follow-up

**Cancer diagnosis**:

Post-transplant cancer

Date of the diagnosis of cancer

Cancer location (ICD-9)

Histopathology

**Follow-up**:

Type of immunosuppressive treatment:

Cyclosporine

Azathioprine

Mycophenolate Mofetil (MMF)

Sirolimus, Everolimus

Tacrolimus

Prednisone

Induction therapy: OKT3, baxiliximab, daclizumab, thymoglobulin and ATG

Dose of immunosuppressive medication

Treatment changes during follow-up

Graft failure

Kidney rejection episodes

New kidney transplants

Death, cause of death

Date of last contact

#### DONOR

Age

Gender

Donor kidney source: cadaveric, living unrelated, living related

HLA mismatches

## Pre-publication history

The pre-publication history for this paper can be accessed here:

http://www.biomedcentral.com/1471-2407/9/294/prepub

## References

[B1] Morales JM Neoplasias y trasplante Nefrología 2006 26 2 12 20

[B2] Campistol JM Minimizing the risk of posttransplant malignancy Transplantation 2009 87 8 Suppl S19 22 19384182 10.1097/TP.0b013e3181a07a57

[B3] Excell L McDonald S Deaths ANZ-DATA Registry 2004 Report Chapter 2004 16 24 http://www.anzdata.org.au/anzdata/AnzdataReport/27thReport/files/Ch03Deaths.pdf

[B4] Buell JF Gross TG Woodle ES Malignancy after transplantation Transplantation 2005 80 2 Suppl S254 264 10.1097/01.tp.0000186382.81130.ba 16251858

[B5] Chapman JR Webster AC Cancer report ANZ-DATA Registry 2004 Report Chapter 10 2004 99 103 http://www.anzdata.org.au/anzdata/AnzdataReport/27thReport/files/Ch10Cancer.pdf

[B6] Chapman JR Webster AC Cancer after renal transplantation: the next challenge Am J Transplant 2004 4 6 841 842 10.1111/j.1600-6143.2004.00486.x 15147414

[B7] Kasiske BL Snyder JJ Gilbertson DT Wang C Cancer after kidney transplantation in the United States Am J Transplant 2004 4 6 905 913 10.1111/j.1600-6143.2004.00450.x 15147424

[B8] Birkeland SA Storm HH Lamm LU Barlow L Blohme I Forsberg B Eklund B Fjeldborg O Friedberg M Frodin L Cancer risk after renal transplantation in the Nordic countries, 1964–1986 Int J Cancer 1995 60 2 183 189 10.1002/ijc.2910600209 7829213

[B9] Jensen P Hansen S Moller B Leivestad T Pfeffer P Geiran O Fauchald P Simonsen S Skin cancer in kidney and heart transplant recipients and different long-term immunosuppressive therapy regimens J Am Acad Dermatol 1999 40 2 Pt 1 177 186 10.1016/S0190-9622(99)70185-4 10025742

[B10] Lindelof B Sigurgeirsson B Gabel H Stern RS Incidence of skin cancer in 5356 patients following organ transplantation Br J Dermatol 2000 143 3 513 519 10971322

[B11] Birkeland SA Lokkegaard H Storm HH Cancer risk in patients on dialysis and after renal transplantation Lancet 2000 355 9218 1886 1887 10.1016/S0140-6736(00)02298-4 10866449

[B12] Kyllonen L Salmela K Pukkala E Cancer incidence in a kidney-transplanted population Transpl Int 2000 13 Suppl 1 S394 398 11112040 10.1007/s001470050369

[B13] Adami J Gabel H Lindelof B Ekstrom K Rydh B Glimelius B Ekbom A Adami HO Granath F Cancer risk following organ transplantation: a nationwide cohort study in Sweden Br J Cancer 2003 89 7 1221 1227 10.1038/sj.bjc.6601219 14520450 PMC2394311

[B14] Vajdic CM McDonald SP McCredie MR van Leeuwen MT Stewart JH Law M Chapman JR Webster AC Kaldor JM Grulich AE Cancer incidence before and after kidney transplantation JAMA 2006 296 23 2823 2831 10.1001/jama.296.23.2823 17179459

[B15] Villeneuve PJ Schaubel DE Fenton SS Shepherd FA Jiang Y Mao Y Cancer incidence among Canadian kidney transplant recipients Am J Transplant 2007 7 4 941 948 10.1111/j.1600-6143.2007.01736.x 17331115

[B16] Grulich AE van Leeuwen MT Falster MO Vajdic CM Incidence of cancers in people with HIV/AIDS compared with immunosuppressed transplant recipients: a meta-analysis Lancet 2007 370 9581 59 67 10.1016/S0140-6736(07)61050-2 17617273

[B17] Hojo M Morimoto T Maluccio M Asano T Morimoto K Lagman M Shimbo T Suthanthiran M Cyclosporine induces cancer progression by a cell-autonomous mechanism Nature 1999 397 6719 530 534 10.1038/17401 10028970

[B18] Maluccio M Sharma V Lagman M Vyas S Yang H Li B Suthanthiran M Tacrolimus enhances transforming growth factor-beta1 expression and promotes tumor progression Transplantation 2003 76 3 597 602 10.1097/01.TP.0000081399.75231.3B 12923450

[B19] Wong G Chapman JR Cancers after renal transplantation Transplant Rev (Orlando) 2008 22 2 141 149 18631867 10.1016/j.trre.2007.12.004

[B20] Kapoor A Malignancy in kidney transplant recipients Drugs 2008 68 Suppl 1 11 19 10.2165/00003495-200868001-00003 18442297

[B21] Dantal J Pohanka E Malignancies in renal transplantation: an unmet medical need Nephrol Dial Transplant 2007 22 Suppl 1 i4 10 10.1093/ndt/gfm085 17456618

[B22] EBPG Expert Group on Renal Transplantation European best practice guidelines for renal transplantation. Section IV: Long-term management of the transplant recipient. IV.6.1. Cancer risk after renal transplantation. Post-transplant lymphoproliferative disease (PTLD): prevention and treatment Nephrol Dial Transplant 2002 17 Suppl 4 31 33 35–36. 10.1093/ndt/17.suppl_4.31 12091638

[B23] EBPG Expert Group on Renal Transplantation European best practice guidelines for renal transplantation. Section IV: Long-term management of the transplant recipient. IV.6.2. Cancer risk after renal transplantation. Skin cancers: prevention and treatment Nephrol Dial Transplant 2002 17 Suppl 4 31 36 12091640

[B24] EBPG Expert Group on Renal Transplantation European best practice guidelines for renal transplantation. Section IV: Long-term management of the transplant recipient IV.6.3. Cancer risk after renal transplantation. Solid organ cancers: prevention and treatment Nephrol Dial Transplant 2002 17 Suppl 4 32, 34–36. 10.1111/j.1600-6143.2007.01908.x 12091641

[B25] Webster AC Craig JC Simpson JM Jones MP Chapman JR Identifying high risk groups and quantifying absolute risk of cancer after kidney transplantation: a cohort study of 15,183 recipients Am J Transplant 2007 7 9 2140 2151 10.1214/aos/1176350951 17640312

[B26] Kalbfleisch JD Prentice RL The statistical analysis of failure time data 2002 New York: Wiley 163 188

[B27] Gray RJ A class of K-sample tests for comparing the cumulative incidente of a competing risk Annals of Statistics 1988 16 1141 1154 10.1214/aos/1176350951

[B28] Fine JP Gray RJ A proportional hazards model for the subdistribution of a competing risk JASA 1999 94 496 509 10.1097/01.TP.0000083897.44391.E8

[B29] The R Project for Statistical Computing. 2.9.0 http://www.r-project.org/

[B30] Epidat. 3.1. Xunta de Galicia, Consellería de Sanidade: Dirección Xeral de Saúde Pública http://dxsp.sergas.es/ApliEdatos/Epidat/cas/default.asp

[B31] Pedotti P Cardillo M Rossini G Arcuri V Boschiero L Caldara R Cannella G Dissegna D Gotti E Marchini F Incidence of cancer after kidney transplant: results from the North Italy transplant program Transplantation 2003 76 10 1448 1451 10.1002/lt.500050106 14657684

[B32] Saeian K Franco J Komorowski RA Adams MB Hepatocellular carcinoma after renal transplantation in the absence of cirrhosis or viral hepatitis: a case series Liver Transpl Surg 1999 5 1 46 49 10.1016/S0140-6736(95)91618-0 9873092

[B33] Stewart T Tsai SC Grayson H Henderson R Opelz G Incidence of de-novo breast cancer in women chronically immunosuppressed after organ transplantation Lancet 1995 346 8978 796 798 10.1016/0190-9622(95)90239-2 7674744

[B34] Euvrard S Kanitakis J Pouteil-Noble C Dureau G Touraine JL Faure M Claudy A Thivolet J Comparative epidemiologic study of premalignant and malignant epithelial cutaneous lesions developing after kidney and heart transplantation J Am Acad Dermatol 1995 33 2 Pt 1 222 229 10.1016/0190-9622(95)90239-2 7622649

[B35] European best practice guidelines for renal transplantation. Section IV: Long-term management of the transplant recipient Nephrol Dial Transplant 2002 17 Suppl 4 1 67 12484407

[B36] Global activity in organ donation and transplantation. IRODAT 2003 Organs, Tissues & Cells 2004 3 159 162

[B37] Otero-Ravina F Romero R Rodriguez-Martinez M Gude F Diaz AI Pita S Gonzalez-Juanatey JR Valdes F Sanchez-Guisande D [Renal transplantation in the northwest of Spain: analysis of the activity in the region of Galicia] Nefrologia 2006 26 2 253 260 16808264

[B38] Wong G Chapman JR Craig JC Cancer screening in renal transplant recipients: what is the evidence? Clin J Am Soc Nephrol 2008 3 Suppl 2 S87 S100 10.2215/CJN.03320807 18309007 PMC3152279

